# Boron Doped Graphene Quantum Structure and MoS_2_ Nanohybrid as Anode Materials for Highly Reversible Lithium Storage

**DOI:** 10.3389/fchem.2019.00116

**Published:** 2019-03-13

**Authors:** Riyanto, Imam Sahroni, Kartick Bindumadhavan, Pei-Yi Chang, Ruey-an Doong

**Affiliations:** ^1^Department of Chemistry, Faculty of Mathematics and Natural Science, Islamic University of Indonesia, Yogyakarta, Indonesia; ^2^Institute of Environmental Engineering, National Chiao Tung University, Hsinchu, Taiwan; ^3^Department of Biomedical Engineering and Environmental Sciences, National Tsing Hua University, Hsinchu, Taiwan

**Keywords:** boron-doped graphene quantum structures (BGQS), MoS_2_, anode materials, reversible capacity, cycling stability

## Abstract

Herein, the boron-doped graphene quantum structure (BGQS), which contains both the advantages of 0-D graphene quantum dot and 2-D reduced graphene oxide, has been fabricated by top-down hydrothermal method and then mixed with molybdenum sulfide (MoS_2_) to serve as an active electrode material for the enhanced electrochemical performance of lithium ion battery. Results show that 30 wt% of BGQS/MoS_2_ nanohybrid delivers the superior electrochemical performance in comparison with other BGQS/MoS_2_ and bare components. A highly reversible capacity of 3,055 mAh g^−1^ at a current density of 50 mA g^−1^ is achieved for the initial discharge and a high reversible capacity of 1,041 mAh g^−1^ is obtained at 100 mA g^−1^ after 50 cycles. The improved electrochemical performance in BGQS/MoS_2_ nanohybrid is attributed to the well exfoliated MoS_2_ structures and the presence of BGQS, which can provide the vitally nano-dimensional contact for the enhanced electrochemical performance. Results obtained in this study clearly demonstrate that BGQS/MoS_2_ is a promising material for lithium ion battery and can open a pathway to fabricate novel 2-D nanosheeted nanocomposites for highly reversible Li storage application.

## Introduction

The rapid technological development and miniaturization of electronic devices need reliably portable and highly efficient energy supply systems. In response to such increasing demands, the present decade has witnessed a thriving interest in development of high performance lithium ion batteries (LIBs). The choice of LIBs is based on their high reliability, user friendliness, safety, and, commendable shelf life for long term usage (Cheng et al., 2017). Since the performance of LIBs are influenced by the electrochemical property of electrodes among many other parameters, there still exists a fast progress on development of superior anode materials (Eftekhari, 2017).

The transition metal dichalcogenides (TMDs) are one of the first families of compound to serve as anode materials in secondary battery because of their gallery-type structure (Whittingham, 1976). The layered structure in such compounds is held by van der Waals force of interaction which acts as the host for intercalation and deintercalation of foreign ions and molecules. Among the TMDs used, molybdenum disulfide (MoS_2_) and its nanocomposites have been used as anode materials for LIBs (Hwang et al., 2011; Cao et al., 2013; Stephenson et al., 2014; Li et al., 2015; Jiang et al., 2016; Teng et al., 2016). The theoretical specific capacity of MoS_2_ is 670 mAh g^−1^ and can be improved by tailoring the number of layers, particle size and morphology during synthesis (Hwang et al., 2011). However, the stacking behavior and the formation of polymeric intermediate decrease the specific capacity and performance of MoS_2_ during the electrochemical cycling, and thereby pose a major challenge in real time application.

More recently, the incorporation of graphene-based nanomaterials such as reduced graphene oxide (rGO), graphene aerogel and graphene nanoflower with MoS_2_ as the anode has been reported to provide an extra volume for Li^+^ ion uptake during charge/discharge, and results in the improved electrochemical performance in comparison with bare MoS_2_ (Cao et al., 2013; Li et al., 2015; Jiang et al., 2016; Teng et al., 2016). The graphitic backbone of graphene family also acts as mechanically buffering matrix to maintain the mechanical integrity of MoS_2_-graphitic carbon nanocomposites during the extended cycle life of LIBs. Teng et al. (2016) have fabricated the vertical MoS_2_ nanosheets over the graphene sheets by hydrothermal treatment and a reversible capacity of 1,077 mAh g^−1^ at 100 mA g^−1^ after 150 cycles was observed. The fabrication of MoS_2_-graphene nanoflower has delivered the reversible capacity of 1,150 and 890 mAh g^−1^ at current density of 0.1 and 1 A g^−1^, respectively (Li et al., 2015). Such a high electrochemical property is mainly attributed to the fact that graphene-based materials prevent the restacking of MoS_2_ and also enable the fast electron kinetics because of their highly conductive and diffusive features (Jiang et al., 2016; Teng et al., 2016). More recently, several studies have used boron-doped rGO for the improvement on the electrochemical performance of LIB as well as the dechlorination of priority pollutants (Bindumadhavan et al., 2017; Sahu et al., 2017). Doping with boron atoms substantially decreases the internal resistance of anode material and increases the defect sites of graphitic structure, which leads to the enhanced electrochemical performance of lithium ion intercalation/deintercalation. Our previous study has shown that the Ag/B-rGO anode material exhibits superior reversible capacity of 1,484 mAh g^−1^ at a current density of 50 mA g^−1^ initially and can retain stably reversible capacity of 430 mAh g^−1^ at 1,000 mA h^−1^ (Bindumadhavan et al., 2017), showing that B-rGO is a promising graphitic-based material for LIBs. However, the combination of B-rGO with MoS_2_ as the anode material for LIB application has received less attention.

In addition to 2-D graphene and 3-D graphene aerogel, the reduction in nanomaterial size is also advantageous on property enhancement. Graphene quantum dots (GQDs), the newly developed 0-D graphene family, have been under limelight because of their exciting surface and electrochemical properties. Currently, various morphologies of carbon based nanomaterials including ordered mesoporous carbons, rGO and GQDs are invariably under the investigation for the development of sensors, supercapacitors, and drug delivery systems (Dutta Chowdhury and Doong, 2016; Liu et al., 2016; Anh et al., 2017; Ganganboina et al., 2017, 2018). The decoration of nitrogen doped-GQDs over the 3-D MoS_2_-rGO nanohybrid has improved the onset potential of oxygen reduction reaction to +0.81 V vs. reversible hydrogen electrode (RHE) (Vinoth et al., 2016). The coral-type MoS_2_/GQD catalyst showed excellent performance with a small onset overpotential of 95 mV and a low Tafel slope for long-term electrocatalytic stability (Guo et al., 2017). Moreover, the GQDs have been used as a component of anode for application in LIB. Guo et al. (2016) have recently investigated the electrochemical performance of MoS_2_/GQD nanocomposites as the anode for LIBs and found that an initial reversible capacity of 1,394 mAh g^−1^ was obtained. However, there remains a large scope to investigate the effect of boron-doped graphene quantum structure (BGQS), which contains both B-GQDs and B-rGO, on the electrochemical performance when combined with MoS_2_ as the anode materials.

**Scheme 1:**
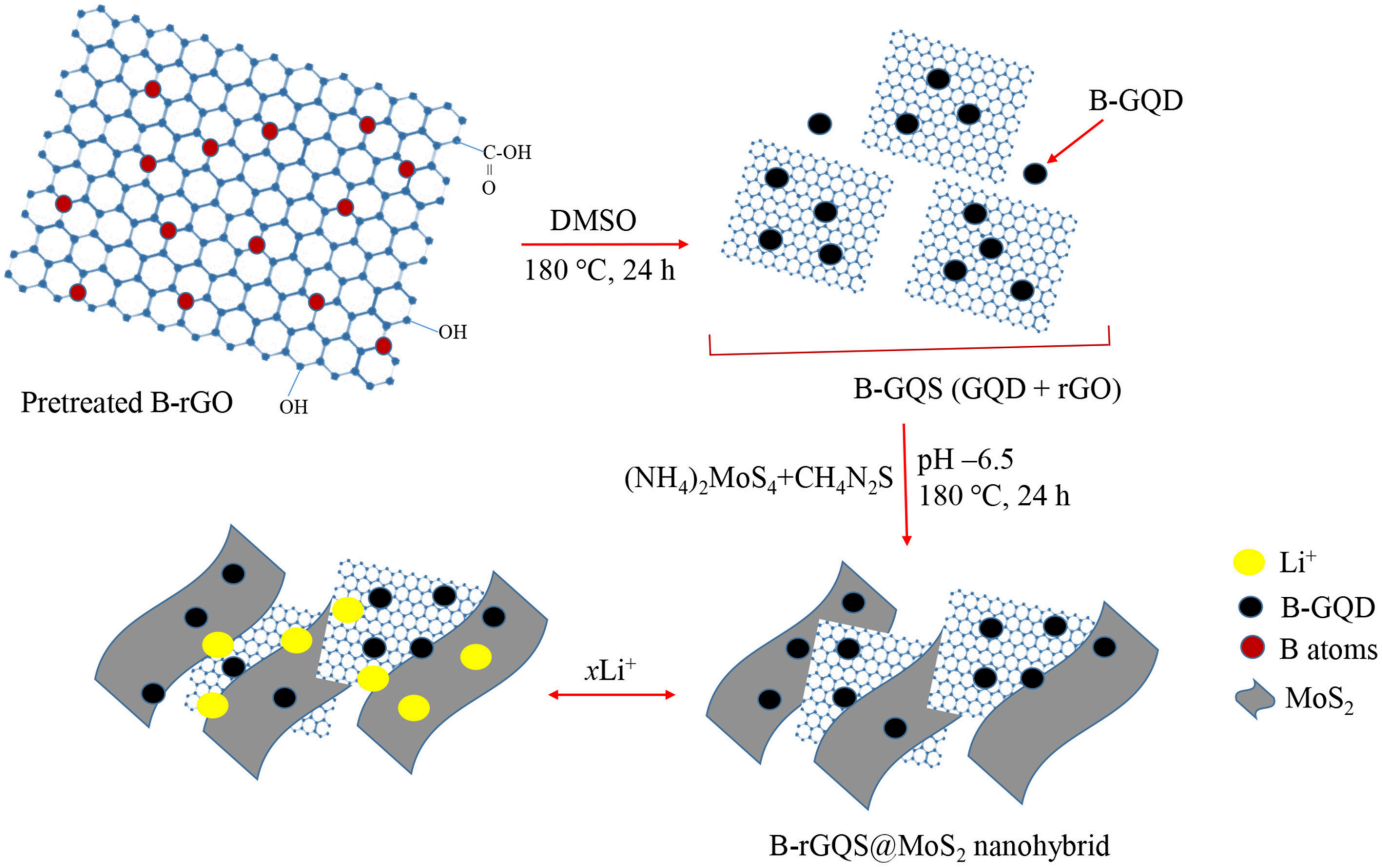
Schematic illustration of preparation of BGQS/MoS_2_ nanohybrids and the application for Li^+^ ion intercalation/deintercalation.

Herein, the BGQS/MoS_2_ with various loadings of BGQS were fabricated to serve as anode materials for LIB application. BGQS, which possesses the advantages of both 0-D B-GQDs and 2-D B-rGO, was first synthesized by top-down hydrothermal method and then 10–70 wt% BGQS were mixed with MoS_2_ as the anode materials for enhanced electrochemical performance of LIBs. As shown in Scheme 1, the fabrication of BGQS involves a single step fragmentation with size reduction under hydrothermal conditions to generate novel B-GQDs embedded onto B-rGO nano-sheeted structures. The *in situ* reduction of molybdate precursor in the presence of BGQS in subsequent hydrothermal reaction at 180°C leads to the formation of BGQS/MoS_2_ nanohybrids. The BGQS/MoS_2_ exhibits excellent electrochemical performance in comparison with bare MoS_2_ and BGQS and the 30 wt% BGQS/MoS_2_ shows superior initial reversible capacity of 3,055 mA g^−1^ at 50 mA g^−1^ with excellent rate capability and cycling stability after 50 cycles. The synergistic effect between BGQS and MoS_2_ improves the electrochemical performance of nanohybrids by reducing the internal resistance as well as acting as nano-dimensional contact points for fast charge transport. Moreover, the BGQS component also serves as a buffering matrix to maintain the mechanical integrity of the anode during charge/discharge processes.

## Materials and Methods

### Preparation of Graphite Oxide

The graphite oxide was prepared by modified Hummers' method by oxidizing pristine graphite with a hard oxidation mixture. In a typical procedure, 100 mg of graphite was mixed with 50 mg NaNO_3_ and 4 mL of concentrated H_2_SO_4_ in an ice bath with stirring. After 30 min of stirring, the mixture was moved to room temperature and 300 mg of potassium permanganate was added gradually, which would result in the change in color from black to deep purple. The stirring was further continued for 1 h to get a thick purple slurry. The above mixture was then diluted by addition of 1 L distilled deionized water (DI water) (18.2 MΩ cm) and allowed to settle. After sedimentation for 48 h, the supernatant was discarded and the remaining yellow solid particles were washed with hot DI water several times, centrifuged, freeze dried, and then stored in desiccator for further analysis.

### Preparation of B-rGO and BGQS

The BGQS was prepared originally from the 2-D rGO. Initially B-rGO was prepared by thermal degradation of 100 mg graphite oxide with 30 wt% boric acid in a quartz tube furnace at 700°C for 4 h under nitrogen atmosphere (Bindumadhavan et al., 2017). The tube furnace was allowed to cool to ambient temperature to form B-rGO. In order to obtain the quantum structures of BGQS by top down approach, B-rGO was again oxidized with a mixture of H_2_SO_4_/HNO_3_ (3/1, v/v) for 24 h. After the treatment, the mixture was centrifuged after repeatedly washed with hot DI water and finally freeze dried. The dry black powder was dispersed in DMSO and hydrothermally heated in Teflon vessel at 180°C for 24 h. The mixture was filtered and washed with DI water after hydrothermal reaction. The BGQS was then obtained as a fine black powder after drying and were then retained for further analysis.

### Fabrication of BGQS/MoS_2_ Nanohybrids

The as-prepared MoS_2_ and BGQS/MoS_2_ nanohybrids were prepared by hydrothermal reaction involving the reduction of ammonium thiomolybdate with thiourea. Aqueous solutions of ammonium thiomolybdate and thiourea were mixed in a molar ratio of 1:2 and at pH 6.5. The mixture was then transferred into a Teflon vessel and the hydrothermal reaction was performed at 180°C for 24 h. Subsequently, the dark gray solid was dried and further calcined under argon gas at 400°C for 2 h, and 250 mg of MoS_2_ was retained after calcination. Moreover, the BGQS/MoS_2_ hybrids were prepared by adding 10, 30, 50, and 70 wt% of BGQS to the identical aqueous solutions of ammonium thiomolybdate and thiourea, which were used in preparation of bare MoS_2_. The obtained hybrids were denoted as BGQS-X/MoS_2_ where X is the loading amount of BGQS.

### Characterization

The structure and morphology of BGQS and BGQS/MoS_2_ nanohybrids were characterized using JEOL JEM-ARM200F transmission electron microscope (TEM) and JEOL JEM-2,010 high-resolution transmission electron microscope (HR-TEM) at an accelerating voltage of 15 and 300 kV, respectively. The morphological images of GQD were also identified using Tecnai G2 F30 S-twin scanning transmission electron microscope (STEM). X-ray photoelectron spectroscopy (XPS) was performed with an ESCA Ulvac-PHI 1,600 photoelectron spectrometer from Physical Electronics using Al Kα radiation photon energy at 1,486.6 ± 0.2 eV. The X-ray diffraction (XRD) patterns were recorded on Bruker D8 X-ray diffractometer with Ni filtered Cu-Kα radiation (λ = 1.5406 Å). Raman spectra of nanomaterials including bare BGQS, MoS_2_ and BGQS/MoS_2_ nanohybrids were recorded with Bruker Senterra micro-Raman spectrometer equipped with an Olympus BX 51 microscope and DU420-OE CCD camera. The thermogravimetric analysis of MoS_2_, BGQS and BGQS/MoS_2_ at various BGQS loadings was determined by thermogravimetric analysis (TGA) using Mettler Toledo DSC/TGA 3+ Stare system in air.

### Electrochemical Measurement

The electrochemical measurement of half cells were performed by mixing 70 wt% of BGQS/MoS_2_ nanohybrids with 20 wt% carbon black and 10 wt% polyvinylidene fluoride in 0.3 mL of *N*- methylpyrrolidone, and then well-mixed in a mortar until a homogeneous slurry was obtained. The slurry was then spread onto a copper foil current collector and dried in vacuum at 60°C for 6 h. The 2,032 type coin cells were assembled in an argon-filled glove box using the coated copper foil as the working electrode, Li metal foil as the counter electrode, and 1.15 M solution of LiPF_6_ in a 1:1:1 (v/v/v) mixture of ethylene carbonate, ethyl methyl carbonate and dimethyl carbonate as the electrolyte. The cells were charged and discharged galvanostatically under the current density range of 50–1,000 mA g^−1^ by a Maccro Model 4,300 battery testing system at room temperature. In addition, cyclic voltammogram (CV) was obtained at a scan rate of 0.1 mV s^−1^ in a fixed voltage window of 0.01–3 V (vs. Li^+^/Li). The electrochemical impedance spectra (EIS) were carried out by an Autolab PGSTAT 302N electrochemical test system (Metrohm Autolab B.V., Netherlands) in a two-electrode system with a sine wave of 10 mV amplitude over a frequency range of 100 kHz to 0.01 Hz.

## Results and Discussion

### Surface Characterization of BGQS/MoS_2_ Nanohybrids

**Figure 1:**
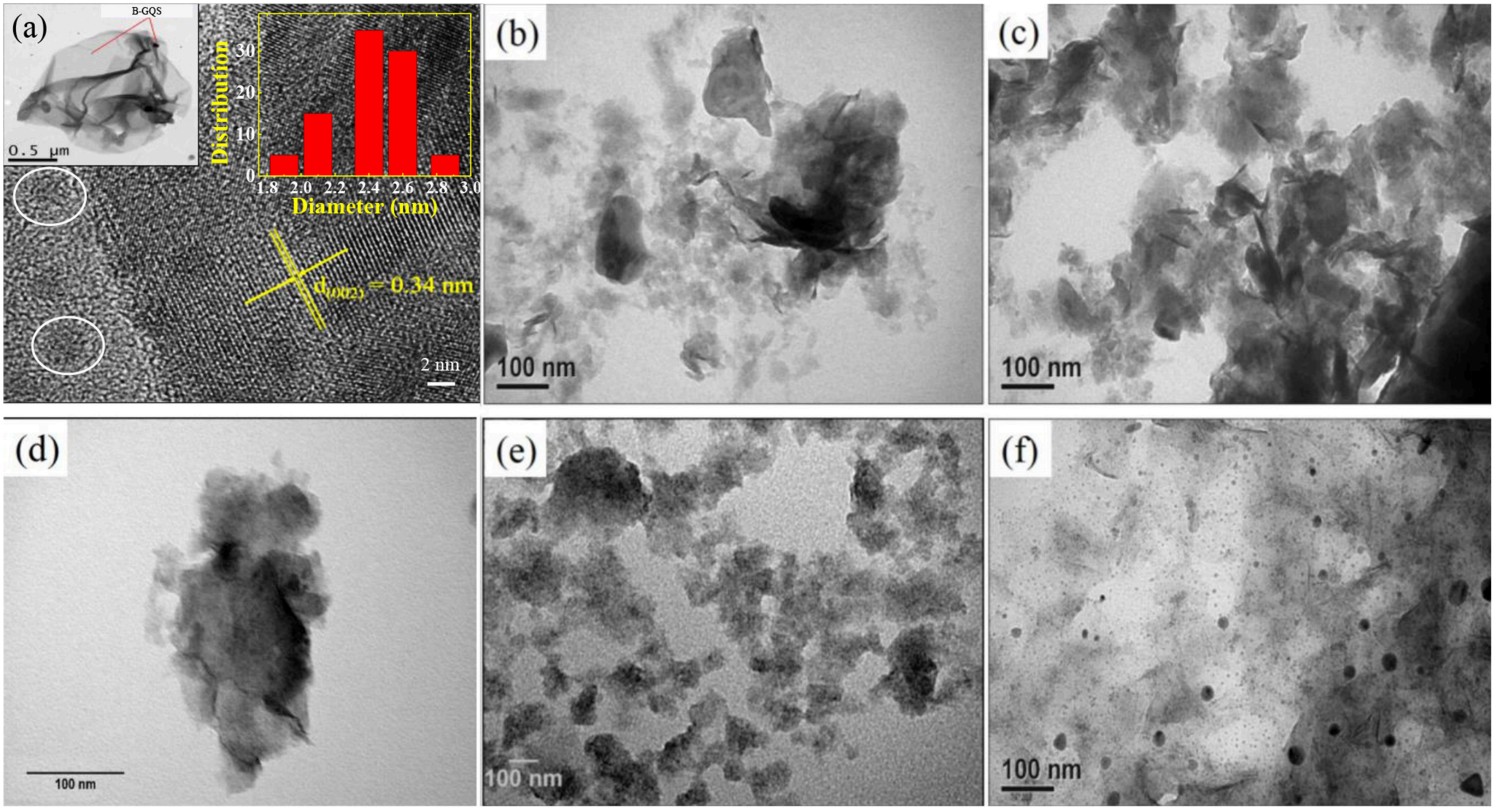
**(a)** STEM images of boron doped graphene quantum structures (BGQS) and TEM images of **(b)** as-prepared MoS_2_, **(c)** BGQS-10/MoS_2_, **(d)** BGQS-30/MoS_2_, **(e)** BGQS-50/MoS_2_, and **(f)** BGQS-70/MoS_2_. Insets of this figure are TEM image (left) and histogram (right) of BGQS. The white circle indicates the particle size of B-GQDs.

The BGQS was fabricated by *in-situ* top-down method from 2-D B-rGO nanosheets and then the morphology of as-synthesized BGQS was first examined by TEM and STEM to elucidate the evolvement of quantum structure. The TEM image of BGQS in the left inset of Figure 1a clearly shows the well-dispersed B-GQDs embedded within the large rGO-based matrix, confirming the formation of twin structures during the fragmentation of B-rGO. The formation of such low dimensional contact points is a result of extensive oxidation during pretreatment and fragmentation under hydrothermal reaction. The developed region of B-GQDs can serve as the attachment center of Li ions to enhance the intercalation/deintercalation capacity for LIB application. In addition, the large domain of B-rGO plays a vital role in acting as a buffering matrix for the enhancement of conductivity and longevity of anodes during the electrochemical charge/discharge cycles. Moreover, the HRTEM image of BGQS (Figure 1) clearly shows the formation of B-GQDs (white circle in Figure 1) after hydrothermal treatment and the fringes of B-GQDs can be well-matched with the (002) diffraction plane of graphene with an interlayer spacing of 0.34 nm, indicating the purity in the crystalline region of as-prepared BGQS. In addition, the particle sizes of B-GQDs are in the range of 1–4 nm with mean particle size of 2.5 nm (right inset of Figure 1), which is in good agreement with the reported data of GQDs (Dutta Chowdhury and Doong, 2016; Ganganboina et al., 2017, 2018).

The TEM images of bare MoS_2_ and BGQS/MoS_2_ nanohybrids with various BGQS loadings of 10–70 wt% were further recorded to assess the change in morphology after inclusion of BGQS. As illustrated in Figure 1b. The as-prepared MoS_2_ appears as the well-exfoliated few layered structures, which are transparent and stable to the electron beam irradiation. After loading with 10 and 30 wt% BGQS, the TEM images show the co-dispersion of both BGQS and MoS_2_ (Figures 1c,d) and 30 wt% BGQS can be clearly attached onto the surface of layered MoS_2_. It is important to note that a severe aggregation of MoS_2_ occurs when 50 wt% of BGQS is mixed with MoS_2_ (BGQS-50/MoS_2_) (Figure 1e), which is a result of excessive interaction from large proportion of BGQS and may result in the loss of electrochemical properties. The TEM image of 70 wt% BGQS/MoS_2_ (BGQS-70/MoS_2_) shows the complete phase separation of BGQS structures as they exist as the individual entity and inhomogenously spread over the thin platelets of MoS_2_ (Figure 1f).

**Figure 2:**
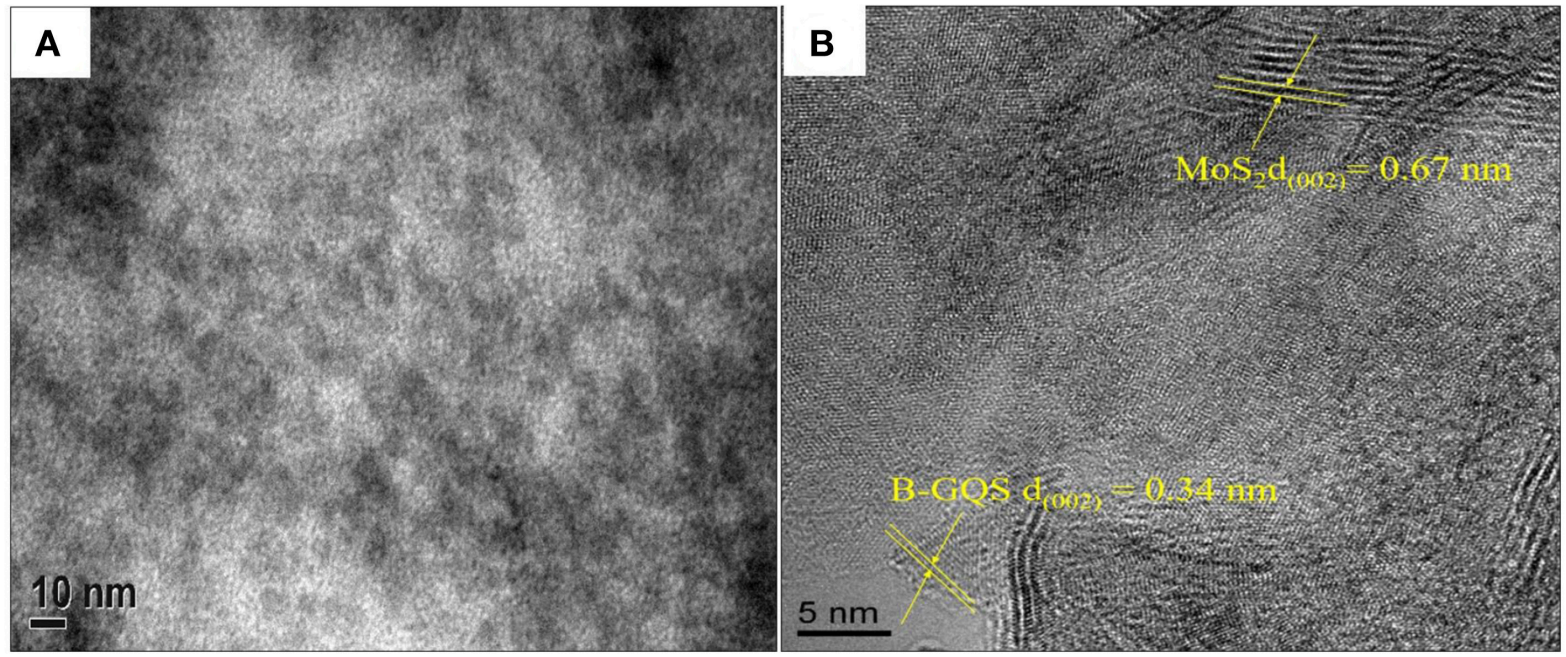
**(A)** HRTEM and **(B)** STEM images of the BGQS-30/MoS_2_ nanohybrid with the corresponding lattice indications of MoS_2_ and BGQS.

Figure 2A shows the HRTEM image of BGQS-30/MoS_2_. The irregularly shaped BGQS can be embedded into the lattice of MoS_2_, which confirms the homogeneous co-dispersion of 30 wt% BGQS in nanohybrids. From the STEM image shown in Figure 2B, the clear fringes of BGQS structures (0.34 nm) and MoS_2_ (0.67 nm) pertain to their characteristic (002) diffraction planes, which indicates the purity of BGQS/MoS_2_ nanohybrids. Such a co-existence of the components is necessary to enhance the charge and electron transport kinetics in the electrochemical application and the homogeneity in BGQS-30/MoS_2_ structure can improve the anodic behavior of MoS_2_-based material for LIB application.

**Figure 3:**
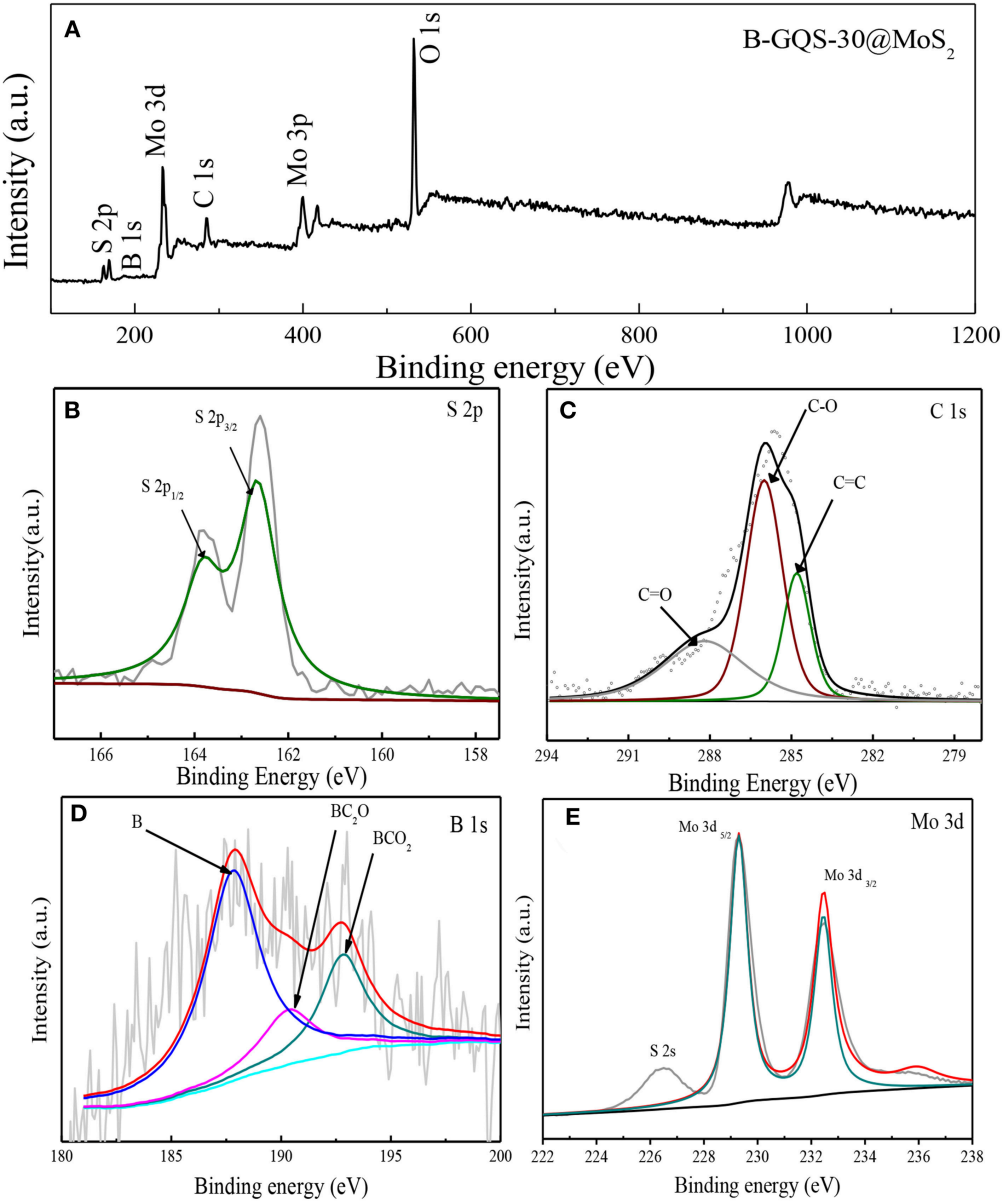
**(A)** XPS survey spectra of BGQS-30/MoS_2_ and the deconvoluted spectra of **(B)** S 2p, **(C)** C 1s, **(D)** B 1s, and **(E)** Mo 3d peaks.

XPS is an effective technique to identify the chemical species of elements in nanomaterials (Chang and Doong, 2004), which was used to determine the chemical environments and the presence of boron element in BGQS/MoS_2_. The 30 wt% BGQS/MoS_2_ was used as the model material because the XPS spectra of various loadings of BGQS/MoS_2_ nanohybrids are similar. As shown in Figure 3A, the survey scan shows the presence of C 1 s, Mo 3 d and S 2 p peaks at 284.5, 232.5, and 169.8 eV, respectively. In addition, a low intensity peak corresponding to B 1 s at 188.9 eV is observed, clearly showing the successful doping of B atoms into BGQS/MoS_2_ nanohybrids. The deconvoluted S peak shows the contribution from S 2p_1/2_ and S 2p_3/2_ centered at 163.7 and 162.6 eV, respectively (Figure 3B). The deconvoluted C 1s peak exhibits C-O, C = O and C = C functionality at 285.9, 288.3, and 284.7 eV, respectively, indicating the presence of oxygenated groups in BGQS structures (Figure 3C). The two deconvoluted B 1s peaks at 190.5 and 192.8 eV are mainly contributed from the chemical bonds of BC_2_O and BCO_2_, respectively (Figure 3D), which confirm the existence of partial bonding between B and sp^2^ hybridized C atoms in the BGQS (Lin et al., 2011). A large peak at 187.8 eV is attributed to the presence of elemental B in BGQS/MoS_2_ nanohybrids. Moreover, the Mo 3d peak after deconvulation shows a doublet of Mo 3d_3/2_ and Mo 3d_5/2_ at 232.4 and 229.3 eV, respectively (Figure 3E), which can be assigned as the characteristic peaks of Mo^4+^ in MoS_2_ (Dong et al., 2015; Wang et al., 2017).

**Figure 4:**
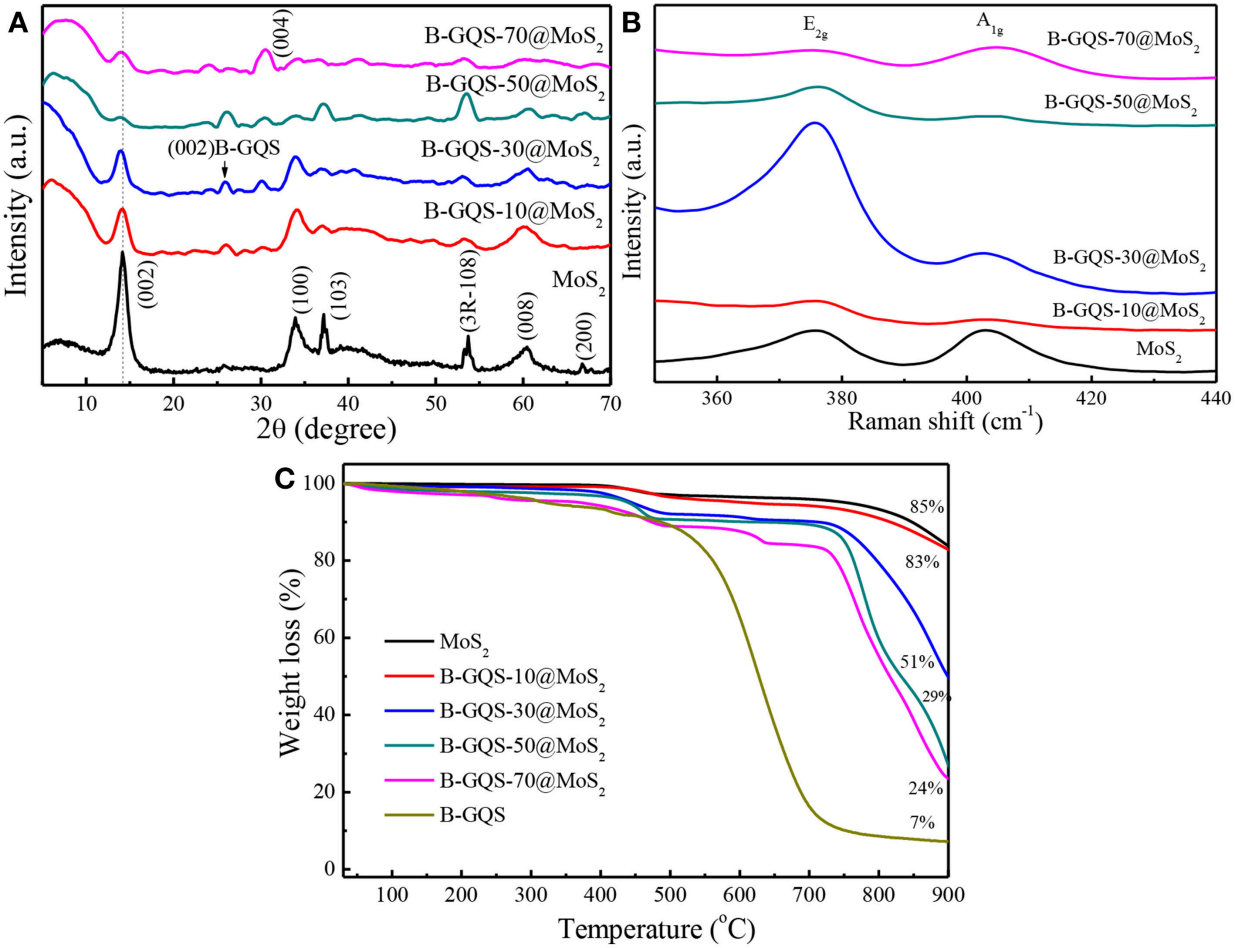
**(A)** XRD patterns, **(B)** Raman spectra, and **(C)** thermograms of MoS_2_ and BGQS/MoS2 nanohybrids with various BGQS loading of 10–70 wt%.

Figure 4 shows the microstructural analysis of MoS_2_, BGQS and their nanohybrids including crystallinity, thermal property and structural fingerprint. The XRD patterns of as-prepared MoS_2_ shows peaks at 2θ = 14.1°, 33.9°, 37.1°, 60.4°, and 66.7°, which correspond to (002), (100), (103), (008), and (200) reflection planes, respectively (JCPDS = 77–1716). An additional peak at 53.6° 2θ belongs to the (108) plane of 3R phase of MoS_2_ (Wei et al., 2016). After addition of various weight ratios of BGQS, the intensity of (002) plane of 2H-MoS_2_ diminishes with the increase in BGQS content, presumably attributed to the loss of layer stacking and formation of small crystallites of MoS_2_. In addition, the (002) reflection peak of MoS_2_ in BGQS-30/MoS_2_ and BGQS-50/MoS_2_ shows a slight shift from 14.1° to 13.9° 2θ, indicating that the addition of 30–50 wt% BGQS particles enhance the layer distortions in 2H-MoS_2_ (Bindumadhavan et al., 2013). Such layer distortion and exfoliation may enhance the intercalation/deintercalation kinetics of Li^+^ ions and molecules during the charge/discharge reactions. However, the (002) reflection of MoS_2_ in BGQS-70/MoS_2_ reappears at 14.06° 2θ along with the increase in strong reflection from (004) plane, depicting the re-stacking of MoS_2_ again at high BGQS loading of 70 wt%. Moreover, the MoS_2_ with 70 wt% BGQS exhibits the phase separation due to the inhomogeneous co-dispersion. It is noteworthy that low peak intensity of (002) plane of BGQS also appears at 26.2° 2θ in all BGQS/MoS_2_ nanohybrids, clearly indicating the presence of graphitic backbone structures of graphene family.

The key change in the structures of BGQS/MoS_2_ after the addition of various ratios of BGQS can be identified by Raman spectra. The as-prepared MoS_2_ shows the presence of two peaks centered at 375.9 and 403.3 cm^−1^ arising from the E_2g_ and A_1g_ vibration modes, respectively (Figure 4B). The opposite vibration of two S atoms with respect to the Mo atoms gives rise to the E_2g_ mode, while A_1g_ mode is attributed to the out-of-plane vibration of only S atoms in opposite direction (Li et al., 2012). The change in E_2g_ and A_1g_ peak intensity also indicates the variation in stacking and layer arrangement in MoS_2_ after the inclusion of BGQS. It is interesting to note that both the E_2g_ and A_1g_ peaks of all nanohybrids appear distinguishably and their peak intensities vary with the change in BGQS contents. The BGQS-30/MoS_2_ nanohybrid shows the maximum enhancement in E_2g_ intensity in comparison with A_1g_ peak, which can be attributed to the formation of less stacked structure (Lee et al., 2010). Moreover, BGQS-30/MoS_2_ exhibits the similar spectral feature to the reported result of layered MoS_2_ (Li et al., 2012) and the E_2g_ and A_1g_ peaks show red and blue shifts, respectively, which occur as a result of optimal exfoliation and few layer stacking. The shift in peaks is highly related to the defects and electronic interactions of MoS_2_ with BGQS entity. The presence of few layered structure of BGQS can be beneficial in improving the intercalation properties of BGQS-30/MoS_2_ nanohybrids for LIB application. The further increase in BGQS loading to 50–70 wt%, however, results in the aggregation of MoS_2_, and subsequent decreases the peak intensity of E_2g_ and A_1g_ by stiffening the vibration modes in opposite direction (Lee et al., 2010). This result supports the fact that 30 wt% BGQS can be exfoliated into few layers with distinct co-dispersion of fine particles, which can lead to superior conductivity of nanohybrids to improve their performance as anode in LIBs.

The thermal stability of MoS_2_ and BGQS/MoS_2_ nanohybrids was evaluated by performing TGA under air atmosphere. As displayed in Figure 4C, the thermogram of MoS_2_ shows the weight loss when temperature >400°C, which is attributed to the oxidation of MoS_2_ to molybdenum oxide (MoO_2_) and sulfur dioxide (SO_2_) (Yang et al., 2016). A total weight loss of 15% for MoS_2_ is observed, showing the high thermal stability of MoS_2_. In contrast, the bare BGQS exhibits the typical thermal behavior of a graphitic material, wherein the weight loss continues from 100 to 700 °C. The slight weight loss at 50–200°C is mainly from the loss of water molecules and then the subsequent decrease in weight at 200–450°C is attributed to the pyrolysis of thermally labile oxygenated functional groups. A nearly complete weight loss in the temperature range of 500–700°C is the exothermal removal of remaining oxygenated moiety and the complete degradation of graphitic carbon backbone (Hsiao et al., 2013). It is noteworthy that the residual weight of 7 wt% for bare BGQS after 900 °C is the doped amount of B atoms in BGQS.

For BGQS/MoS_2_ nanohybrids, the thermal stability reduces with the increase in mass loading of BGQS and a two-stage decomposition is observed for BGQS-10/MoS_2_. In the first stage of thermal degradation, small amount of the physisorbed water molecules as well as oxygenated functional groups are lost up to 400°C, and then follows the oxidation of MoS_2_ and final breakdown of BGQS backbone, which is similar to that of bare MoS_2_. However, a distinct three-stage decomposition can be noted when the loading amount of BGQS is in the range of 30–70 wt%. The slight weight loss at 50–400°C is mainly from loss of physisorbed water molecules and oxygenated functional groups of carbon materials. The second stage of weight loss between 400 and 750°C can be attributed to the oxidation of MoS_2_ and BGQS structures. Moreover, the obvious weight loss at temperature >750°C is the degradation of residual graphitic backbone structures inhibited by MoS_2_ to provide any further thermal stability (Thangappan et al., 2016).

### Electrochemical Performance of BGQS/MoS_2_

**Figure 5:**
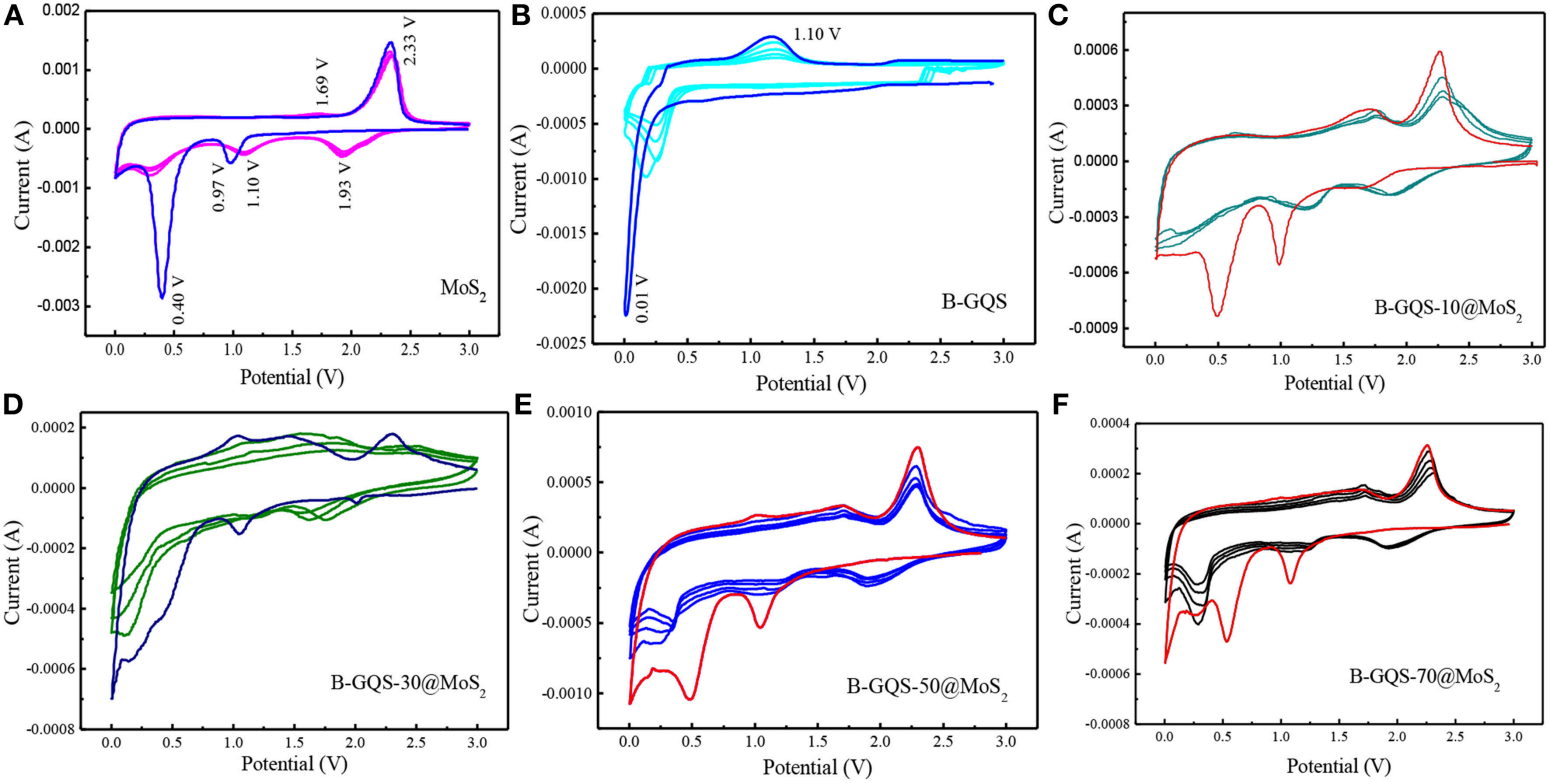
Cyclic voltammograms of MoS_2_, BGQS and BGQS/MoS_2_ nanohybrids with various BGQS loadings of 10-70 wt%. **(A)** Pure MoS2, **(B)** pure BGQS, **(C)** 10 wt%, **(D)** 30 wt%, **(E)** 50 wt%, and **(F)** 70 wt%.

The electrochemical properties of BGQS/MoS_2_ with respect to lithium ion intercalation-deintercalation were first evaluated by the CV curve for five cycles at a scan rate of 0.1 mV s^−1^ in the potential window of 0.01 to 3.0 V. The CV curves of few layered MoS_2_ exhibit several well-defined characteristic peaks, which match well with the finding of reported MoS_2_ (Xiao et al., 2011; Wang et al., 2014). As shown in Figure 5A, two prominent peaks at 0.97 and 0.4 V are observed during the first cathodic cycle. The peak at 0.97 V is the insertion reaction of Li ions to MoS_2_ (Equation 1), leading to the formation of Li_x_MoS_2_ along with the phase transformation of MoS_2_ from trigonal prismatic (2H) to octahedral (1T) lithiated MoS_2_ (Wang et al., 2014). It is noteworthy that the peak appeared at 0.4 V is intensive in the first cycle but decreases dramatically in the subsequent cathodic curves of 2–5 cycles, which indicates the irreversible intercalation reaction of Li_x_MoS_2_ and Li ions to form Li_2_S and Mo (Equation 2). Moreover, the formation of metallic Mo (Equation 2) can significantly enhance the conductivity of the whole electrode (Xiao et al., 2011), and subsequently enhances the electrochemical performance of B-QGS/MoS_2_ nanohybrids.

(1) $${\text{MoS}}_{2}+\text{x}{\text{Li}}^{+}+\text{x}{\text{e}}^{-}\to {\text{Li}}_{\text{x}}{\text{MoS}}_{2}$$

(2) $${\text{Li}}_{\text{x }}{\text{MoS}}_{2}+(\text{4}-\text{x }){\text{Li}}^{+}+(4-\text{x }){\text{e}}^{-}\to 2{\text{Li}}_{2}\text{S}+\text{Mo}$$

In the subsequent cathodic cycles, less intense peak at 1.93 V is observed, indicating the generation of Li_2_S from MoS_2_, while another peak at 1.10 V is the continuous reaction of Mo with Li. During the anodic scans of 1st−5th cycles, the strong peak at 2.33 V is mainly attributed to the delithiation of Li_2_S during the charging process (Wang et al., 2013). Moreover, a dormant peak at 1.69 V can be assigned to the oxidation of metallic Mo. The highly overlapped CV curves of 2nd−5th cycles mean the good reversibility of BGQS/MoS_2_ for intercalation/deintercalation of Li^+^ ions.

The CV curves of bare BGQS were also recorded in order to elucidate its contribution to lithium uptake. As presented in Figure 5B, the anode composed of bare BGQS shows a steady trough in the first cathodic cycle between 0.9 and 0.5 V, which can be attributed to the formation of solid electrolyte interface (SEI) layer produced from the reaction between BGQS and electrolyte. A strong peak arising from the reaction and uptake of Li ions by the basal planes of BGQS is observed at 0.01 V. It is important to note that this peak shifts to 0.16–0.26 V with the significant decrease in peak intensity in the subsequent 2nd−5th cathodic cycles, indicating the occurrence of an irreversible Li ions uptake. In the first anodic cycle, the appearance of peak at 1.10 V is the delithiation of Li ions from BGQS structures to form LiC_6_ structures (Bindumadhavan et al., 2017). However, the decrease in peak intensity at 1.10 V in the 2nd−5th anodic cycle is the irreversible uptake of Li ions by bare BGQS. After incorporation of various amounts of BGQS with MoS_2_, the CV curves of all the BGQS/MoS_2_ nanohybrids resemble the features of bare MoS_2_ (Figures 5C–F). In addition, the nanohybrids with BGQS loading of >30 wt% show the contribution from BGQS component in the potential window of 0.01–0.3 V, depicting that both MoS_2_ and BGQS can exhibit good electrochemical performance in nanohybrids.

**Figure 6:**
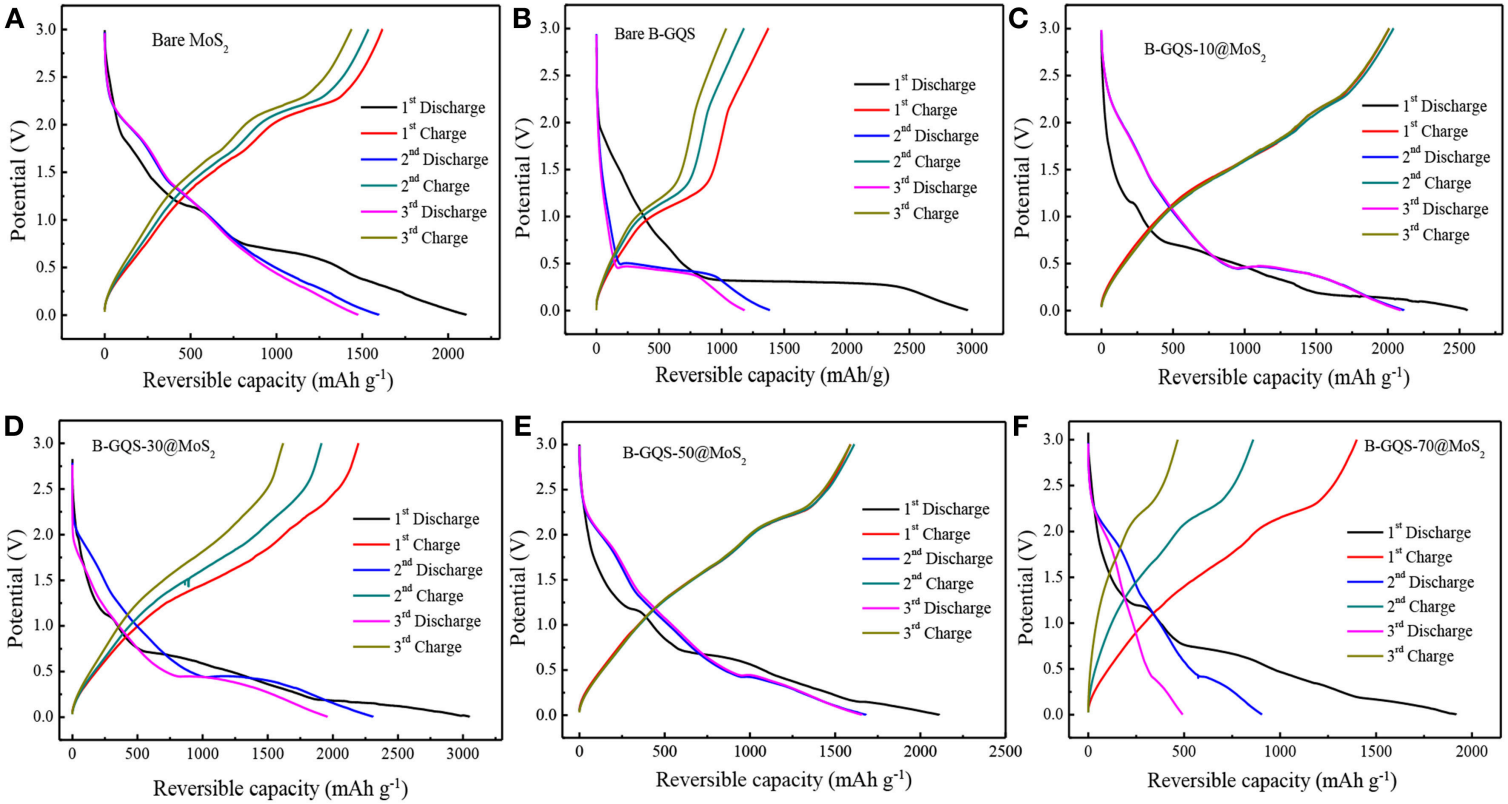
The glvanostatic discharge-charge curves of **(A)** bare MoS2, **(B)** bare BGQS **(C)** BGQS-10/MoS2, **(D)** BGQS-30/MoS2, **(E)** BGQS-50/MoS2, and **(F)** BGQS-70/MoS2 nanohybrids at a current density of 50 mA/g in the potential window of 0.01–3.0 V.

Figure 6 shows the first three cycles of galvanostatic charge-discharge curves (GCD) of BGQS/MoS_2_ nanohybrids at a current density of 50 mA g^−1^ in the potential window of 0.01–3.0 V. The bare MoS_2_ shows the plateaus located at 1.13 and 0.67 V in the first discharge curve (Figure 6A). The plateau appeared at high potential is ascribed to the lithium ion intercalation into the MoS_2_ lattices, resulting in the formation of Li_x_MoS_2_. Furthermore, the conversion reaction of Li_x_MoS_2_ to Mo particles embedded in Li_2_S is characterized by the plateau at 0.67 V (Ma et al., 2014). The slope continues to decrease below 0.5 V, which indicates the formation of SEI layers along with the electrochemical degradation of electrolyte and lithium storage at the interfaces of Li_2_S and Mo phases (Huang et al., 2013). The obvious change in the GCD curves during the 2nd and 3rd cycles is also observed in the discharge curves where the plateau occurs in the potential range of 2.1–1.9 V and 1.3–1.1V, respectively, which is in good agreement with the CV curves. Similar to the discharge curve, the plateau at 2.1–2.3 V in the initial charge curve is attributed to the deintercalation of Li ions and incomplete oxidation of Mo (Huang et al., 2013). It is noteworthy that the bare MoS_2_ exhibits a highly reversible capacity of 2,106 mAh g^−1^ in the first discharge, and a corresponding charge capacity of 1,621 mAh g^−1^ is obtained. The loss in coulombic efficiency of 23% in the first cycle can be attributed to the irreversible lithium uptake and the formation of SEI layers. From the second cycle onwards, a 96–99% of columbic efficiency is observed, indicating the good cycling characteristics of bare MoS_2_.

The charge-discharge curves of BGQS were also recorded to investigate its lithium uptake characteristic with respect to applied potential. As shown in Figure 6B, the first discharge curve of BGQS is similar to the profile of typically mesoporous carbon materials reported elsewhere (Vinoth et al., 2016), wherein the gradual slope up to 0.5 V is attributed to the Li ion uptake majorly by the basal planes and defect sites over the structures of BGQS. The reversible capacity of BGQS in the voltage range of 0.5–0.3 V is the direct intercalation of Li ions to the BGQS layer interfaces and the extended slope beyond 0.2 V is ascribed to the additional storage capacity of lithium ions in the basal planes of BGQS. It is also noted that the slight decrease in slope at the potential of <0.5 V is attributed to the formation of SEI layer, leading to the irreversible capacity (Chang and Chen, 2011). In contrast, a plateau due to the delithiation reaction is noted at 1.1 V in the first discharge curve. The first discharge capacity of bare BGQS is 2,996 mAh g^−1^ with a columbic efficiency of 46%. This low columbic efficiency of bare BGQS in the first cycle can be attributed to the formation of large proportion of SEI layers. Furthermore, the BGQS also shows obvious capacity loss in the subsequent charge-discharge cycles, possibly due to the fact that the presence of 0-D GQD particles is unable to retain their structural stability during volume expansion in charge-discharge reaction.

Figures 6C–F show the GCD curves of all the BGQS/MoS_2_ nanohybrids with various BGQS loadings of 10–70 wt%. The combination of MoS_2_ with 10–30 wt% BGQS exhibits a synergistic effect on the electrochemical performance. The initial discharge capacity of BGQS/MoS_2_ increases from 2,554 mAh g^−1^ for BGQS-10/MoS_2_ to 3,061 mAh g^−1^ for BGQS-30/MoS_2_. However, the increase in BGQS loading to 50–70 wt% decreases the reversible capacity to 1,917–2,110 mAh g^−1^. It is important to note that the first discharge profiles of all the nanohybrids are similar to the characteristic curve of bare MoS_2_, which the plateaus located at 1.13 and 0.67 V are clearly observed. However, a slight difference in the first charge profile of BGQS-30/MoS_2_ is noted where the plateau at 2.1–2.3 V is unclear. This indicates the formation of small MoS_2_ particles. Moreover, the well-dispersed BGQS in 30 wt% BGQS/MoS_2_ nanohybrids makes the conversion reaction more reversible in comparison with the bare MoS_2_. The initial discharge capacity of 3,061 mAh g^−1^ with a corresponding charge capacity of 2,203 mAh g^−1^ for BGQS-30/MoS_2_ is delivered, implicating a 72% of columbic efficiency for the initial capacity. However, the BGQS-30/MoS_2_ nanohybrid shows a high retention in columbic efficiency after 2nd discharge-charge cycles, depicting the superior electrochemical properties. In addition, the decreased reversible capacity of BGQS/MoS_2_ at 50–70 wt% BGQS is attributed to the phase separation of the components due to aggregation of BGQS and MoS_2_, resulting in the inefficient electrochemical reaction with Li ions.

**Figure 7:**
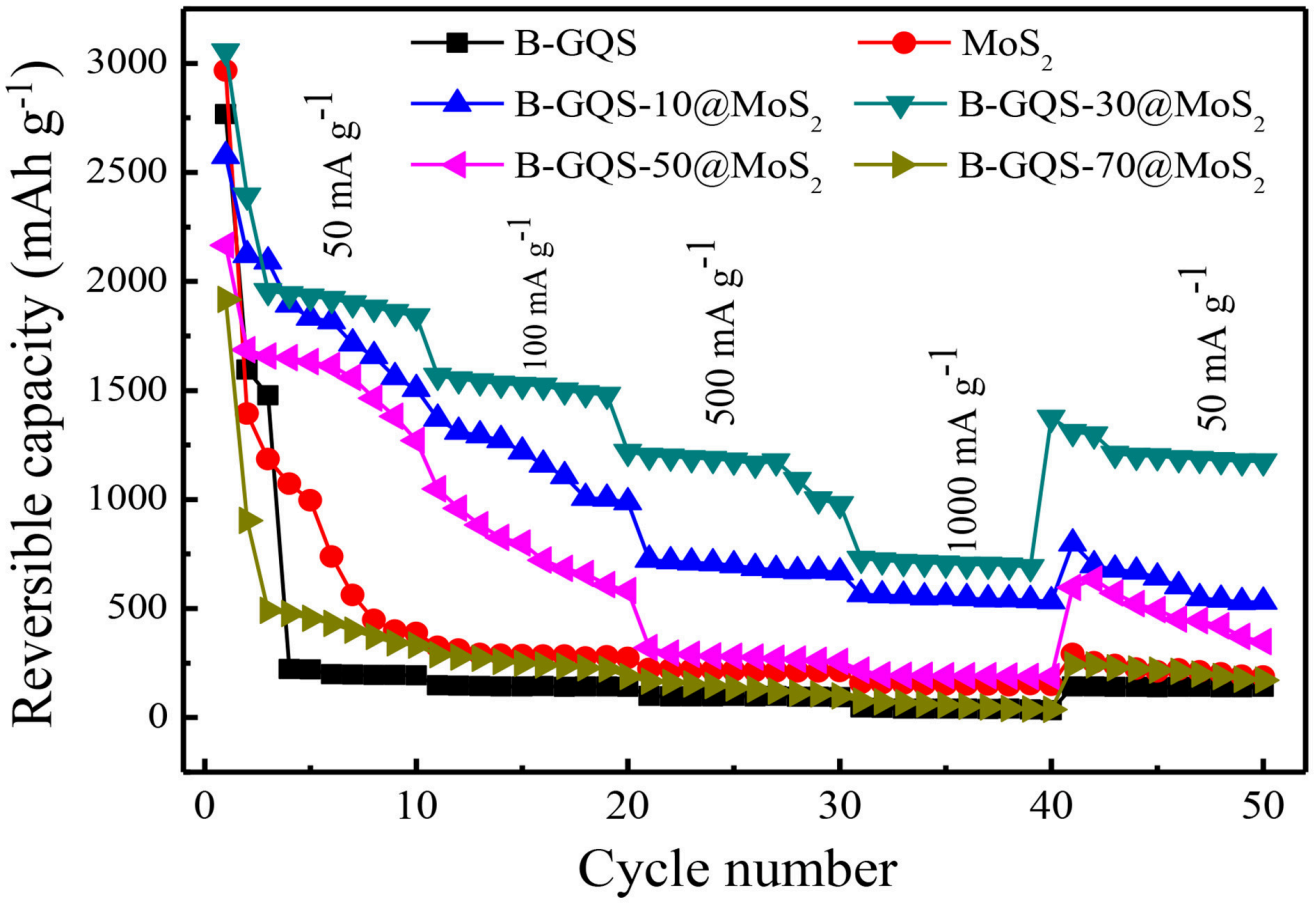
Rate capability of MoS_2_, BGQS and BGQS/MoS_2_ nanohybrids at various current densities ranging from 50 to 1,000 mA g^−1^.

Figure 7 displays the rate capability of BGQS/MoS_2_ nanohybrids fabricated with 10–70 wt% BGQS at various current densities ranging from 50 to 1,000 mA g^−1^. In addition, the reversible capacity of BGQS/MoS_2_ is compared with those of as-prepared MoS_2_ and BGQS. The bare MoS_2_ exhibits a high Li^+^ ion uptake capacity with initial reversible discharge capacity of 2,966 mAh g^−1^ at a current density of 50 mA g^−1^. The high reversible capacity of MoS_2_ at low current density is probably due to the well-exfoliated and few-layered MoS_2_ obtained in this study, which has been shown in TEM image. However, the reversible capacity of MoS_2_ fades dramatically with the increase in the first 10 cycles at 50 mA g^−1^ and only 387 mAh g^−1^ is obtained after 10 cycles, which is mainly attributed to the decomposition of electrolytes. The reversible capacity continues to decrease when the current density increases and only 187 mAh g^−1^ is obtained at 1,000 mA g^−1^ because of the low mechanical strength and structural integrity of bare MoS_2_.

Moreover, the reversible capacity of bare MoS_2_ does not recover to its original capacity when the current density is back to 50 mA g^−1^. At the end of 50 cycles, only reversible capacity of 187 mAh g^−1^ is obtained, which is 48% of the original discharge capacity after 10 cycle at 50 mA g^−1^. The poor performance can be attributed to the serious aggregation of MoS_2_ layers and the formation of SEI layers between the 2-D porous structures, making the kinetics of lithium uptake sluggish. Similarly, the initial reversible capacity of BGQS is 2,766 mAh g^−1^ at 50 mA g^−1^ and then fades dramatically after the first 5 cycles, possibly due to the formation of SEI layers and irreversible reaction between Li^+^ and BGQS. The capacity of BGQS is 220 mAh g^−1^ during the subsequent cycles at 50 mA g^−1^ and further decreases upon increasing the current density. At a high current density of 1,000 mA g^−1^, BGQS exhibit a reversible capacity of merely 36 mAh g^−1^, suggesting that the BGQS structure as an individual component is not a suitable anode material for retaining a high amount of Li ions during intercalation.

The combination of MoS_2_ with BGQS improves the stability of rate capacity at high current density. The BGQS-10/MoS_2_ nanohybrid shows an exceedingly improved rate performance compared to its individual components at all current densities of 50–1,000 mA g^−1^ and maintains a relatively high reversible capacity of 575 mAh g^−1^ at 1,000 mA g^−1^. Among all the nanohybrids prepared in the present study, the BGQS-30/MoS_2_ nanohybrid exhibits superior rate capability to other BGQS/MoS_2_ nanohybrids. The BGQS-30/MoS_2_ delivers an excellent initial discharge capacity of 3,055 mAh g^−1^ at a current density of 50 mA g^−1^ and retains the reversible capacity of 1,523 mAh g^−1^ at 100 mAh g^−1^. When the current density further increases to 500 and 1,000 mA g^−1^, the reversible capacity is noted to be 1,175 and 715 mAh g^−1^, respectively. Apart from the high reversible capacity at various current densities, the BGQS-30/MoS_2_ also exhibits an excellent electrochemical performance in capacity when the current density is returned to 50 mA g^−1^ and the reversible capacity is recovered to 1,374 mAh g^−1^ at the 40th cycle. At the end of 50th cycle, a high reversible capacity of 1,175 mAh g^−1^ is retained, exhibiting its superior electrochemical performance compared to those individual components (bare MoS_2_ and BGQS) and other nanohybrids.

The robust electrochemical performance of BGQS-30/MoS_2_ is attributed to the optimal co-dispersion among the components, resulting in a synergistic effect on electrochemical performance. The HRTEM image of BGQS-30/MoS_2_ (Figure 2) clearly shows the homogenous embedment of BGQS onto the MoS_2_ layers. The presence of BGQS provides additional intercalation sites for lithium ions, which is also evident from the CV curves. It is also important to note that the presence of 0-D GQD structures embedded in 2-D rGO and/or MoS_2_ provides active sites for strong attachment of lithium ions during discharge, which can improve their uptake capacity. In addition, the quantum structures act as nano-dimensional contact points for fast electron and charge transport during the electrochemical cycling (Guo et al., 2016). The graphitic backbones also act as the volume buffering matrix, which significantly improve the mechanical stability of anode materials during the continuous expansion and contraction of MoS_2_ layers during charge-discharge cycles (Chang and Chen, 2011).

The further increase in weight percent of BGQS to 50–70 wt% decreases the electrochemical performance on rate capacity at various current densities. The reversible capacity of BGQS-70/MoS_2_ at all current densities lies between those of bare MoS_2_ and BGQS. Such a loss in capacity retention at high weight loading of BGQS is mainly attributed to the phase separation of BGQS and MoS_2_, and subsequently lowers the uptake capacity of Li ions. In addition, the MoS_2_ layers tend to restack at high BGQS content, resulting in the loss of mechanical stability as well as the decrease in availability on transport pathways for the intercalation and deintercalation of lithium ions and molecules. This sluggish kinetics leads to the loss in stable reversibility of lithium charge/discharge rates at high BGQS loading.

**Figure 8:**
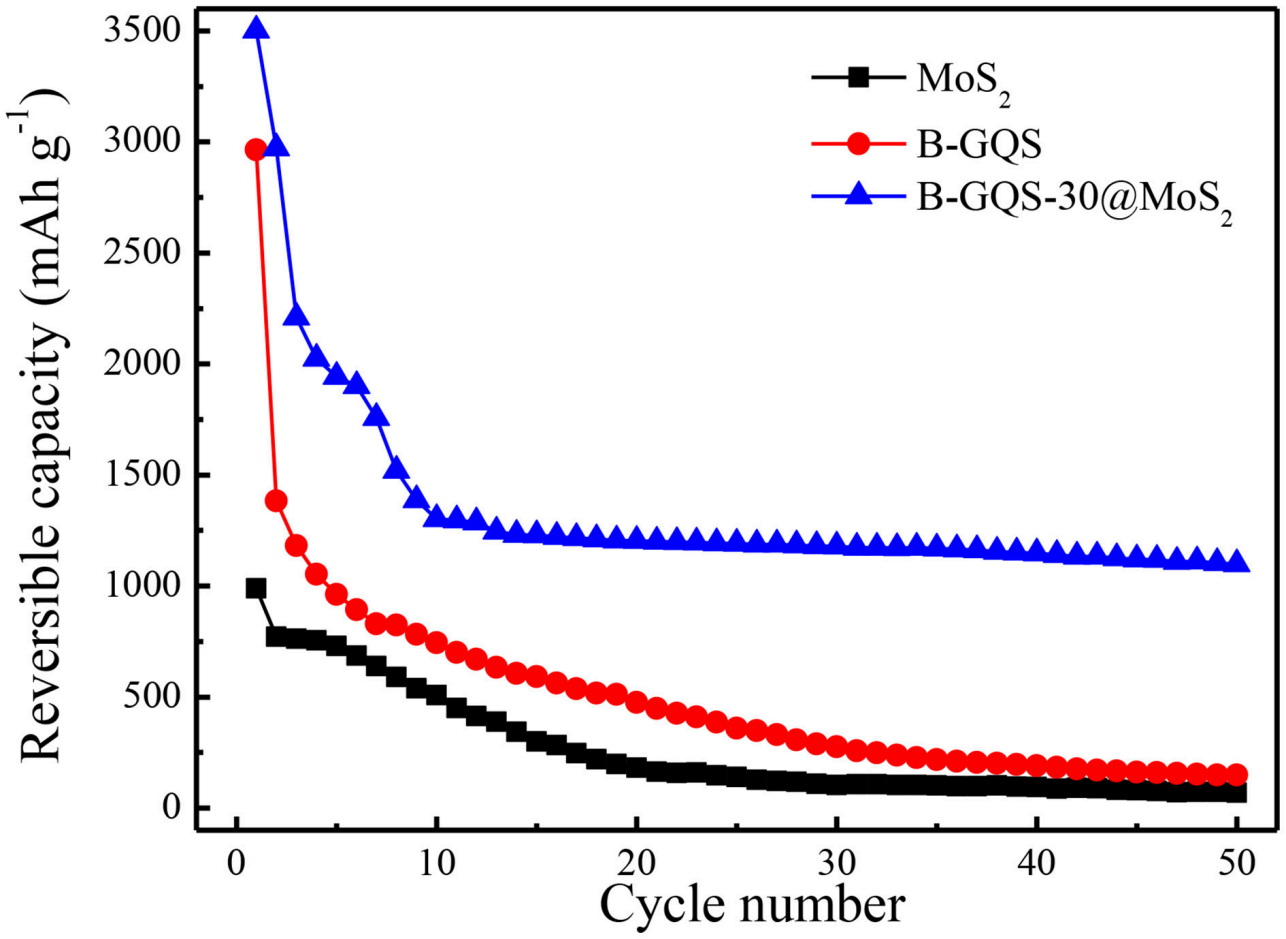
Cycling stability performance of MoS_2_, BGQS and BGQS-30/MoS_2_ at 100 mA g^−1^.

In order to examine the improvement on the long-term performance for application, the electrochemical cycling stability of BGQS-30/MoS_2_ was performed. Figure 8 shows the electrochemical performance of BGQS-30/MoS_2_ nanohybrids at a current density of 100 mA g^−1^ for 50 cycles. The bare MoS_2_ and BGQS show a high reversible capacity of 1,015 and 2,964 mAh g^−1^, respectively, at the initial cycle. However, the reversible capacity of both bare 2-D nanomaterials decreases significantly during the subsequent cycles and only 10–15% of their initial capacity is retained at the end of 50 cycles. The BGQS-30/MoS_2_ nanohybrid exhibits the synergistic effect on the enhancement of the long-term electrochemical performance, and an initial reversible capacity of 3,491 mAh g^−1^ is obtained. Although the capacity of BGQS-30/MoS_2_ decreases during the first 10 cycles because of the formation of SEI layers and side reactions, the reversible capacity reach a stable condition and a reversible capacity of 1,041 mAh g^−1^ is maintained after 50 cycles. Wang et al. fabricated the single layered MoS_2_-graphene nanosheet nanocomposites for LIB application and a capacity of 571 mAh g^−1^ at 1,000 mA g^−1^ was obtained (Wang et al., 2013). A previous study combined GQDs with MoS_2_ and an initial discharge capacity of 1,394 mAh g^−1^ at a current density of 100 mA g^−1^ was reported (Guo et al., 2016). These results clearly indicate that the electrochemical performance of BGQS-30/MoS_2_ is exceedingly superior in comparison with the individual bare MoS_2_ and BGQS and other reported data (Wang et al., 2013; Li et al., 2015; Guo et al., 2016; Jiang et al., 2016; Teng et al., 2016; Bindumadhavan et al., 2017). The significant enhancement in cycling stability can be attributed to the optimal deposition loading of BGQS with few layered MoS_2_ sheets to restrict the agglomeration of MoS_2_ species and maintain the capacity retention for a long cycle life. The presence of BGQS can act as buffering sites during the extended cycling and provide required volume for absorption of mechanical stress developed during the intercalation/deintercalation (Chang and Chen, 2011; Huang et al., 2013).

## Conclusions

In this study, we have demonstrated the superiority of BGQS/MoS_2_ as the high-performance anode material for LIB application. The top-down strategy can successfully prepare BGQS by embedding 0-D GQD onto a large 2-D rGO fragment. The morphological and structural characterization confirm the formation of well-dispersed few layered BGQS onto MoS_2_ nanohybrids to improve the conductivity as well as to provide nano-dimensional contact points for the enhanced uptake capacity of Li ions. BGQS-30/MoS_2_ is an excellent anode material for highly reversible Li storage and stable rate capability. An excellent initial discharge capacity of 3,055 mAh g^−1^ is achieved by BGQS-30/MoS_2_ nanohybrid at a current density of 50 mA g^−1^. The BGQS-30/MoS_2_ nanohybrid also exhibits the superior rate capability and a highly reversible capacity of 715 mAh g^−1^ at a current density of 1,000 mA g^−1^ is obtained. In addition, the reversible capacity of 1,041 mAh g^−1^ at 100 mA g^−1^ after 50 cycles is retained, clearly indicating the excellent long cycle life because the presence of BGQS can serve as the buffering matrix to adsorb the mechanical stress during intercalation/deintercalation processes. Results obtained in this study clearly demonstrate that BGQS/MoS_2_ is a promising anode material which can open an avenue to fabricate novel B-doped GQS/MoS_2_ nanocomposites for highly reversible Li storage application.

## Author Contributions

All authors listed have made a substantial, direct and intellectual contribution to the work, and approved it for publication.

### Conflict of Interest Statement

The authors declare that the research was conducted in the absence of any commercial or financial relationships that could be construed as a potential conflict of interest.
